# Non-linear interactions between candidate genes of myocardial infarction revealed in mRNA expression profiles

**DOI:** 10.1186/s12864-016-3075-6

**Published:** 2016-09-17

**Authors:** Katherine Hartmann, Michał Seweryn, Samuel K. Handleman, Grzegorz A. Rempała, Wolfgang Sadee

**Affiliations:** 1 College of Medicine Center for Pharmacogenomics, The Ohio State University Wexner Medical Center, Biomedical Research Tower, 460 W 12th Avenue, Columbus, OH USA; 2 Department of Molecular Virology, Immunology, and Medical Genetics, The Ohio State University, Biomedical Research Tower, 460 W 12th Avenue, Columbus, OH USA; 3 Faculty of Mathematics and Computer Science, University of Łodz, Łodz, Poland; 4 Division of Biostatistics, College of Public Health, The Ohio State University, 250 Cunz Hall, 1841 Neil Avenue, Columbus, OH USA; 5 Mathematical Biosciences Institute, The Ohio State University, Jennings Hall 3rd Floor, 1735 Neil Avenue, Columbus, OH USA

**Keywords:** RNA expression, Dynamic interactions, Co-expression network, Myocardial infarction, Coronary artery disease

## Abstract

**Background:**

Alterations in gene expression are key events in disease etiology and risk. Poor reproducibility in detecting differentially expressed genes across studies suggests individual genes may not be sufficiently informative for complex diseases, such as myocardial infarction (MI). Rather, dysregulation of the ‘molecular network’ may be critical for pathogenic processes. Such a dynamic network can be built from pairwise non-linear interactions.

**Results:**

We investigate non-linear interactions represented in mRNA expression profiles that integrate genetic background and environmental factors. Using logistic regression, we test the association of individual GWAS-based candidate genes and non-linear interaction terms (between these mRNA expression levels) with MI. Based on microarray data in CATHGEN (CATHeterization in GENetics) and FHS (Framingham Heart Study), we find individual genes and pairs of mRNAs, encoded by 41 MI candidate genes, with significant interaction terms in the logistic regression model. Two pairs replicate between CATHGEN and FHS (CNNM2|GUCY1A3 and CNNM2|ZEB2).

Analysis of RNAseq data from GTEx (Genotype-Tissue Expression) shows that 20 % of these disease-associated RNA pairs are co-expressed, further prioritizing significant interactions. Because edges in sparse co-expression networks formed solely by the 41 candidate genes are unlikely to represent direct physical interactions, we identify additional RNAs as links between network pairs of candidate genes. This approach reveals additional mRNAs and interaction terms significant in the context of MI, for example, the path CNNM2|ACSL5|SCARF1|GUCY1A3, characterized by the common themes of magnesium and lipid processing.

**Conclusions:**

The results of this study support a role for non-linear interactions between genes in MI and provide a basis for further study of MI systems biology. mRNA expression profiles encoded by a limited number of candidate genes yield sparse networks of MI-relevant interactions that can be expanded to include additional candidates by co-expression analysis. The non-linear interactions observed here inform our understanding of the clinical relevance of gene-gene interactions in the pathophysiology of MI, while providing a new strategy in developing clinical biomarker panels.

**Electronic supplementary material:**

The online version of this article (doi:10.1186/s12864-016-3075-6) contains supplementary material, which is available to authorized users.

## Background

Large scale Genome-Wide Association Studies (GWAS) have revealed numerous candidate risk alleles for complex disorders, such as myocardial infarction (MI) and coronary artery disease (CAD) [1, 2]. However, genetic risk of disease at the population level cannot be accounted for by individual genetic variants in single genes or even by summing the individual effects of dozens of genes – a gap often referred to as the ‘missing heritability’ [3, 4]. Thus far, epistasis has also failed to account for heritability [5, 6]. Other factors may involve additional loci not detectable by GWAS, non-additive interactions between candidate genes, and external factors modulating gene expression and interactions [7, 8]. Here, we consider the potential of non-additive effects to contribute to disease risk. Instead of restricting the analysis to genetic variants, we focus on RNA expression levels that integrate multiple factors including genetic differences influencing expression, gene-gene interactions, feedback mechanisms, and environmental influence.

Previous studies of differential mRNA expression in MI and related phenotypes have identified ~1,500 individual genes, but less than 5 % replicate across more than one study [9–16]. Poor replication is often attributed to differences in cohorts and methodology between studies, but also reflects the fact that disease relevant signals are dispersed across many interacting genes (i.e. a network) and that gene expression varies greatly across individuals with different genetic and environmental backgrounds. Low reproducibility of differentially expressed genes limits the biological interpretation and utility of these findings.

Using cluster analysis, GO enrichment analysis, and a variety of machine-learning approaches, studies of differential expression in MI have also implicated particular pathways (e.g. caspase cascade, apoptosis signaling) and cell types (e.g. CD71+ erythroid, NK cells) [9–16]. Efforts however have not yet been made to quantify the non-additive effects of gene interactions that could be revealed from RNA expression patterns varying in the context of MI. Dynamic non-additive interactions could represent essential elements since complex diseases likely do not arise from single perturbations but rather a dysregulation of the molecular network. Indeed, Wu et al. find pairwise non-linear interactions to be important for disease classification and biomarker development [17].

In the present paper, we demonstrate that non-additive, dynamic effects embedded in mRNA expression may play an essential role in defining the odds of a complex phenotype. Focusing on myocardial infarction (MI), we select a well-defined, small number of GWAS-derived candidate genes to probe mechanisms inaccessible on a genome-wide scale. Using whole blood expression arrays from the CATHeterization GENetics Study (CATHGEN) and the Framingham Heart Study (FHS), we test the association of RNA expression profiles with MI. We focus first on individual genes and then expand to consider non-linear interaction terms between pairs of candidate genes.

Analysis of differential co-expression between RNAs can enhance our understanding of how dynamic feedback mechanisms between pairs and defined networks of mRNAs determine disease risk [18–21]. Gene networks can be extracted from existing databases, (e.g., Ingenuity Pathway Analysis, KEGG, etc.). However, most databases are generated from mining previously published literature and are thus biased towards those pathways most studied and often neglect tissue specificity and other nuances of gene-gene interactions.

A comprehensive approach to tissue-specific gene-gene interactions, termed NetWAS was recently published [22]. NetWAS uses Bayesian classification based on a vast database of prior knowledge and integration of open-access expression datasets to define tissue-specific interactions. The results confirm the role of well-established genes in select pathways and include novel discoveries. However, with use of linear measures of association and an additive model thresholded by the effects of individual genes, crucial information on dynamic interactions remains hidden. In contrast, the present study focuses on expression patterns to test dynamic interactions relevant to disease. The approach does not quantify the biological likelihood of any interaction based on prior knowledge, but rather evaluates how non-additive effects change the association between mRNA expression and odds of disease risk.

Despite progress in the field of network biology, existing methods do not fully account for variability in co-expression across individuals with different genetic and environmental backgrounds, even in those cases where the underlying method is tailored to detect non-linear dependency patterns. To overcome this limitation, we employ a resampling procedure that generates a quantitative measure of the stability of co-expression across individuals.

Thus, to inform study non-linear pairwise interactions associated with MI, we construct small-scale, tissue specific co-expression networks with candidate genes in healthy individuals using data from blood and tibial artery in GTEx. Analysis of blood profiles supports biomarker discovery, and artery, as the site of atherosclerotic plaque formation characteristic of CAD, captures disease-relevant physiology. We hypothesize that the relevance of a non-linear interaction for disease will be reflected by a difference in both the strength and variability of co-expression between cases and controls, rather than a binary (presence-absence) switch in co-expression. Accordingly, we expect connections between genes in the co-expression network to be the same in both diseased and healthy individuals but their strength and variability to change between those with and without MI.

Non-linear interactions between candidate genes associated with MI are unlikely to represent direct, physical interactions between mRNAs but rather distinct biological processes that are coordinately regulated. Thus, we expand co-expression networks beyond candidate genes to identify additional mRNAs that may serve to mediate the observed interactions.

## Results

### Differentially expressed individual candidate genes and their interactions in myocardial infarction (MI)

We analyzed RNA profiles (measured by expression arrays in blood) from CATHGEN and the Framingham Heart Study in subjects with and without a history of MI. Focusing on established candidate genes, we searched for: (1) individual of mRNA transcripts and (2) interactions between mRNA transcripts, significantly associated with MI status, noting those that replicate between the two cohorts.

#### GWAS based candidate genes in MI

The CAD Genome Wide Association Study performed by the CARDIOGRAMplusC4D consortium published in 2014, including >60,000 cases and 130,000 controls, identified 45 loci associated with myocardial infarction and CAD at genome-wide significance (Additional file 1) [1, 2]. Forty-one of the candidate genes assigned to these loci had one or more corresponding probes on expression arrays used in both CATHGEN (89 probes) and the Framingham Heart Study (41 probes). All probes corresponding to these genes were tested for an association between expression and MI using logistic regression.

#### Expression of individual candidate genes in MI

The approach for detecting differentially expressed RNAs was designed for each study separately as CATHGEN and FHS include different proportions of cases/controls, levels of relatedness, and racial diversity.

##### CATHGEN

**Fig. 1:**
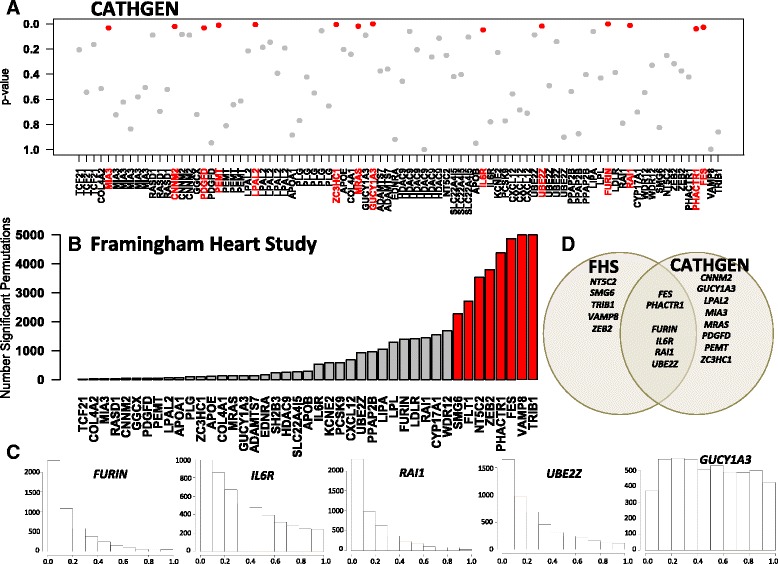
Differentially expressed candidate genes in MI. **a** CATHGEN. *P*-value of association between expression of probe ID (labeled by assigned gene) and MI status from logistic regression with age, race, and gender as additional covariates. Genes with *p*-value less than 0.05 are colored in red. **b** Framingham Heart Study. Number of resamples (of total 5000) in which mRNA expression of GWAS-based candidate gene was significantly associated with MI (*p* < 0.05) in conditional logistic regression model. Controls were matched to cases on age, gender, and belonging to a different family. TRIB1, VAMP8, FES, PHACTR1, ZEB2, NT5C2, and SMG6 (colored in red) had expression profiles strongly associated with MI (i.e. median *p*-value among bootstrap replicates < 0.05). **c** Histograms of *p*-values for genes determined as significant in CATHGEN that did not meet the criteria for being associated with MI in FHS. FURIN, IL6R, RAI1, and UBE2Z were informative of MI based on right-skewed histogram. **d** Venn diagram displaying overlap between genes individually significant in CATHGEN and the Framingham Heart Study

We measured the association between expression of each individual probe, assigned to the 41 candidate genes, and MI status using logistic regression with age, race, and gender as additional covariates in the model (*n* = 1250; 359 cases and 891 controls). We identified 14 candidate genes with at least one probe displaying expression levels nominally (*p* < 0.05) associated with MI: PEMT, RAI1, LPAL2, PDGFD, FES, ZC3HC1, PHACTR1, CNNM2, GUCY1A3, UBE2Z, MRAS, FURIN, IL6R, MIA3 (Fig. 1a, Additional file 1).

**Fig. 2:**
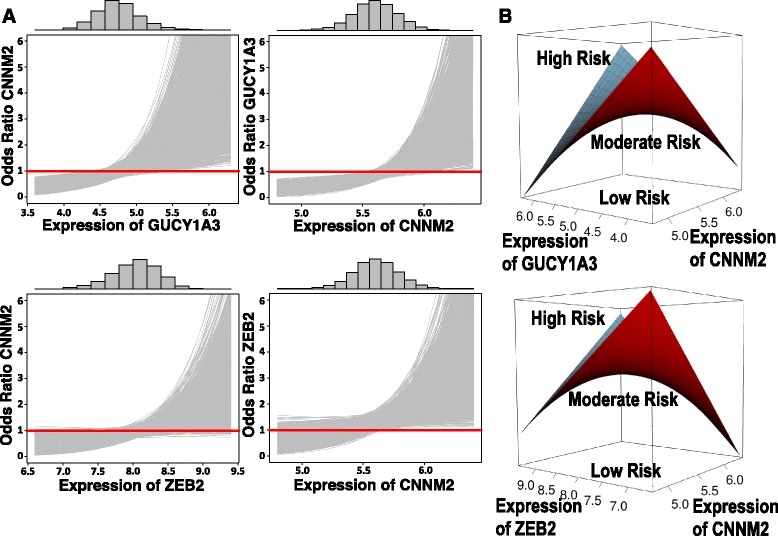
Odds ratios and conditional odds ratios of MI for non-linearly interacting pairs. **a** Conditional odds ratios of MI from one standard deviation increase in the mean expression of gene A, plotted against expression of the second interacting gene, gene B. Histograms of gene B expression are displayed above each panel. The odds ratio is defined as:*odds(Ex_A + sd(Ex_A))/odds(Ex_A)* where *Ex_A* and *sd(Ex_A)* denote the expression level and the standard deviation of expression of gene A respectively. Red lines indicate an odds ratio of 1. In panel **b** the interaction is displayed as surface plot, which is a three dimensional plot of expression of the two genes *versus* the log-odds of MI. The curvature of the saddle shape surface indicates the magnitude of the interaction term in the model

##### Framingham Heart Study

As a population-based cohort, FHS had a significantly smaller prevalence of MI; accordingly we used a matched case–control design. MI cases with expression data available (*n* = 193) were matched to controls (selected from a pool of 4952 subjects) by age, gender, and different family assignment. To assess the robustness of association between mRNA levels and MI status, conditional logistic regression analysis was performed 5000 times, each time on a different random set of matched controls. We identified eight candidate genes displaying expression levels nominally (*p* < 0.05) associated with MI in half or more of the 5000 resamples (i.e. in which the sample median of the *p*-values was less than 0.05): TRIB1, VAMP8, FES, PHACTR1, ZEB2, NT5C2, FLT1, and SMG6 (Fig. 1b, Additional file 1), with PHACTR1 and FES replicating from CATHGEN.

Our resampling approach results in a distribution of main effects and *p*-values that reflects the biological and environmental diversity of cases and controls. We evaluated these distributions in FHS for all genes significant in CATHGEN. With a right-skewed distribution, probes in FURIN, IL6R, RAI1, and UBE2Z were considered to be informative of MI (Fig. 1c). With approximately uniformly distributed *p*-values, probes in CNNM2, GUCY1A3, MRAS, MIA3, PDGFD, PEMT, and LPAL2 did not show evidence of differential expression with MI in FHS (Additional file 2). Uncertainty in correctly assigning differentially expressed genes indicates that single mRNA expression profiles in peripheral blood are only moderately informative of MI status but still can serve as a basis for further analysis.

### Pairs of interacting candidate genes

#### Expression of candidate gene pairs in MI

In addition to searching for individual differentially expressed genes, we considered pairs of mRNAs with and without an interaction term in the model. The expression profiles of mRNA pairs were evaluated for association with MI, using the same approach applied to individual mRNA expression levels in CATHGEN and in Framingham.

##### CATHGEN

Considering an additive logistic model (logit(MI) ~ RNA1 + RNA2 + additional covariates) revealed 56 (of 3916) pairs of probes with both RNA terms as significant (*p* < 0.05) and 1064 with only one RNA term as significant in the model (for details of the model, see Methods section). These 1120 pairs of probes include only 20 of the 40 candidate genes. Applying the same criterion of significance to an interactive model (logit(MI) ~ RNA1 + RNA2 + RNA1*RNA2 + additional covariates) revealed 53 (of 3916) pairs of probes with both RNA terms as significant (*p* < 0.05) and 624 with only one RNA term as significant. These 677 pairs include 38 of the 40 candidate genes. We find an interaction model reveals fewer pairs of RNAs that are comprised of a more diverse set of genes (Table 1).

**Table 1:** Comparison of additive models with and without non-linear interactions between candidate gene pairs in CATHGEN

	Additive Model	Interactive Model
Number of pairs	1120	677
Number of candidate genes	20	38

Number of significant pairs defined as having one or both RNAs associated with MI (*p* < 0.05) and number candidate genes forming those pairs. Shown for an additive *versus* interactive model. Additive model: MI as explained by main effects of candidate genes (logit(MI) ~ RNA1 + RNA2 + additional covariates). Interactive model: MI as explained by main effects of candidate genes and an interaction term (logit(MI) ~ RNA1 + RNA2 + RNA1*RNA2 + additional covariates)

We define a pair of RNAs to be interacting non-linearly if we detect a statistically significant (*p* < 0.05) interaction term in the logistic model for this pair (regardless of the significance of individual RNA terms as evaluated above). By this definition, we found 167 (of 3916) pairs of probes defined as interacting in CATHGEN. These probe pairs represent 149 gene pairs that include nearly all of the candidate genes (40 of 41) (Additional file 3).

##### Framingham Heart Study

As before, we performed conditional logistic regression 5000 times on different sets of matched cases and controls, and recorded the number of times each term in the model was significant (*p* < 0.05). We analyzed both the additive model accounting for MI with expression of gene A and B (MI ~ gene A + gene B) and the model with an additional interaction term (MI ~ gene A + gene B + gene A*gene B). We identified 6 of 903 possible pairs with strong evidence of a non-linear interaction between the two candidate genes (sample median of *p*-values for the interaction term less than 0.05): CNNM2|GUCY1A3, CNNM2|PEMT, CNMM2|ZEB2, IL6R|LPAL2, MIA3|SLC22A5, MIA3|ZC3HC1. For each of these six pairs, adding the interaction term to the model greatly decreased the median *p*-values for all terms in the model, suggesting a functionally significant dynamic interaction in the context of MI (Table 2). Two pairs: CNNM2|GUCY1A3 and CNNM2|ZEB2 were also observed in CATHGEN.

**Table 2:** Interactions between candidate gene pairs in the Framingham Heart Study

	Percent significant resamples				
	Additive Model		Interaction Model		
	Gene1	Gene2	Gene1	Gene2	Gene1* Gene2
CNNM2|GUCY1A3	1 %	2 %	89 %	89 %	86 %
CNNM2|PEMT	1 %	1 %	54 %	54 %	54 %
CNNM2|ZEB2	0 %	75 %	56 %	51 %	56 %
IL6R|LPAL2	9 %	1 %	56 %	59 %	60 %
MIA3|SLC22A5	1 %	5 %	84 %	75 %	84 %
MIA3|ZC3HC1	1 %	2 %	58 %	45 %	57 %

Percent of significant resamples (*p* < 0.05) for each term in 5000 repetitions of conditional logistic regression. Additive model: MI as explained by main effects of candidate genes (logit(MI) ~ RNA1 + RNA2). Interactive model: MI as explained by main effects of candidate genes and an interaction term (logit(MI) ~ RNA1 + RNA2 + RNA1*RNA2)

Of the genes forming these six pairs, only ZEB2 and IL6R were also significant by themselves (Table 2 and Fig. 1). The others were not differentially expressed in the Framingham Heart Study, but 7 of the remaining 8 (CNNM2, GUCY1A3, PEMT, LPAL2, MIA3, and ZC3HC1) were differentially expressed in CATHGEN as individual RNAs. Detectable overlap between CATHGEN and FHS supports the finding of non-linear interactions relevant for MI.

#### MI-relevance of non-linear interacting mRNA pairs

We further investigated how these non-linear RNA interactions affect the odds of MI by generating sample distributions of effect sizes based on the resampling procedure used in the FHS. For the two pairs that replicated between CATHGEN and FHS (CNNM2|GUCY1A3 and CNNM2|ZEB2) we used the distributions of effect sizes to calculate the odds ratio of MI given that the expression of gene A increases by one standard deviation as a function of expression of gene B (for details see Methods section). In both cases we observe that the expression of one gene (e.g. CNNM2) appears protective when expression of the other (e.g. GUCY1A3) is low and deleterious when it is high (Fig. 2). The relevance of these non-linear interactions in accounting for MI status is reflected by both: (1) the reproducibility of the odds ratio curve between different replicates (i.e. all grey lines in the sample distribution of odds ratios fall within the same region, Fig. 2a), and (2) the saddle surface of the expression *versus* log-odds 3D plot (Fig. 2b). In Fig. 2a, a histogram of gene expression across the population is presented to further assess the utility of this RNA as a biomarker of MI. We observe strongly protective or deleterious mRNA-mRNA ratios in a limited portion of the overall population; nevertheless, the use of multiple gene-gene pairs has potential utility in biomarker panels. The question remains of how these pairs relate to one another.

**Fig. 3:**
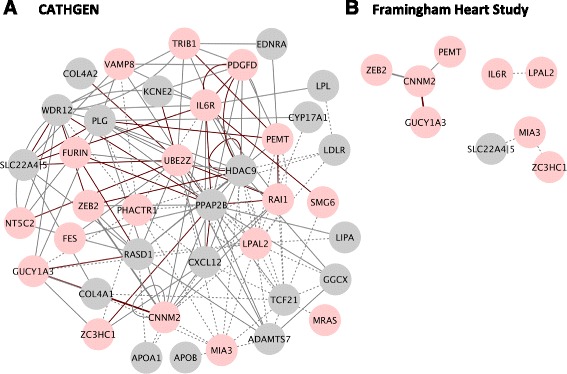
Differentially expressed candidate gene pairs in MI. Nodes are genes and edges reflect significant non-linear interactions defined by logistic models (see Methods). Pink nodes indicate genes that are individually significant (see Fig. 1), red lines indicate gene pairs that are co-expressed (see Fig. 4), dotted lines indicate gene pairs that were not tested for co-expression due to poor expression of one or more of the genes, bolded edges indicate non-linear interactions identified in both cohorts (CNNM2|GUCY1A3; CNNM2|ZEB2). **a** CATHGEN. Connected graph formed by pairs of genes with a significant interaction term in CATHGEN using a logistic model (logit(MI) ~ RNA1 + RNA2 + RNA1*RNA2 + additional covariates). **b** Framingham Heart Study. Disconnected graphs formed by pairs of genes exhibiting statistically significant interaction terms in FHS, defined by means of the bootstrapped conditional logistic regression (logit(MI) ~ RNA1 + RNA2 + RNA1*RNA2) with 5000 repetitions and matching procedure the same as for individual genes

**Fig. 4:**
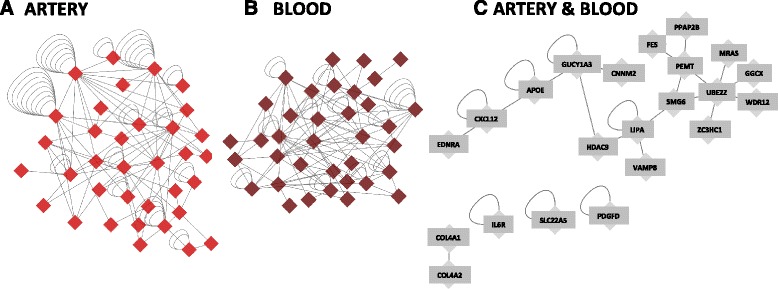
Tissue-specific co-expression networks. Nodes are genes and edges reflect co-expression defined by our algorithm (see Methods). Co-expression of candidate genes in artery (**a**), blood (**b**), and both artery and blood (**c**) show similar structure between the two tissues with little overlap in particular gene pairs. Edges within a single gene indicate co-expression between isoforms of the same gene. Note: not all expressed isoforms are co-expressed

#### Connectivity between non-linear interacting pairs

We investigate the relationship between pairs of non-linearly interacting RNAs by presenting them as a graph of interactions. Pairs with significant interaction terms form a connected graph, which is denser for CATHGEN than Framingham, likely because of the larger number of cases in CATHGEN (Fig. 3a). The high connectivity of the graph of non-linearly interacting RNAs supports the hypothesis that complex phenotypes arise as a destabilization of feedback within a dense molecular network. The six pairs in FHS do not form a densely connected graph, but do indicate interactions between genes only indirectly connected in the CATHGEN network (Fig. 3b).

It appears that the selected 41 candidate genes are informative but support only sparsely populated physiological networks. Therefore, we proceeded to co-expression analysis to both assess the biological plausibility that these genes are co-regulated and incorporate additional RNAs that could serve as linkers (relays) between the 41 candidate genes.

### Co-expression of candidate genes and inclusion of additional genes complementing sparse networks

#### Co-expression patterns among 41 candidate genes

We used co-expression in healthy individuals to assess the biological plausibility of non-linear interactions associated with disease by building small co-expression networks for the 41 candidate genes using GTEx RNAsequencing data from ‘healthy’ individuals in whole blood (*n* = 190) and arteries (*n* = 137). (Fig. 4).

##### Network construction procedure

Our algorithm is designed to evaluate the stability of co-expression between pairs of RNAs, rather than the strength of the co-expression *per se*. Similar to the ARACNE procedure our method uses an information-theoretic divergence measure (Renyi divergence) to detect ‘direct’ non-linear dependencies between expression profiles [23, 24]. To quantify the stability of ‘dynamically co-expressed’ pairs, we generate networks on multiple (50,000) random subsamples of individuals and create a consensus network where weights on the edges between mRNAs are defined by the proportion of subsamples where the two mRNA transcripts are observed as co-expressed (see Additional files 4 and 5 and Methods section). 50 % of all possible pairs are observed in at least one of the 50,000 resamples. For further analysis, we focus on those observed in at least half of the 50,000 resamples, representing only 1 % of all possible pairs (for rationale see Methods section).

##### Disease Interactions and Co-expressions

Twelve (11 %) of the 108 non-linearly interacting pairs in CATHGEN were co-expressed in artery and 21 (19 %) in blood, indicating the potential for coordinated regulation of these mRNAs (Additional files 3 and 6). Those gene pairs that were co-expressed in blood were more likely to demonstrate non-linear interactions associated with MI as manifested by a greater specificity in MI prediction among co-expressed pairs (Additional file 7). One of the non-linear interacting pairs detected in both CATHGEN and FHS (CNNM2|GUCY1A3) was found to be dynamically co-expressed in both artery and blood (Fig. 4). CNNM2|GUCY1A3 was co-expressed in 51 % of the 50,000 resamples in artery and 53 % in blood. These results suggest co-expression patterns determined in healthy individuals are also relevant for disease.

#### Co-expression patterns among candidate genes in networks including additional gene transcripts

**Fig. 5:**
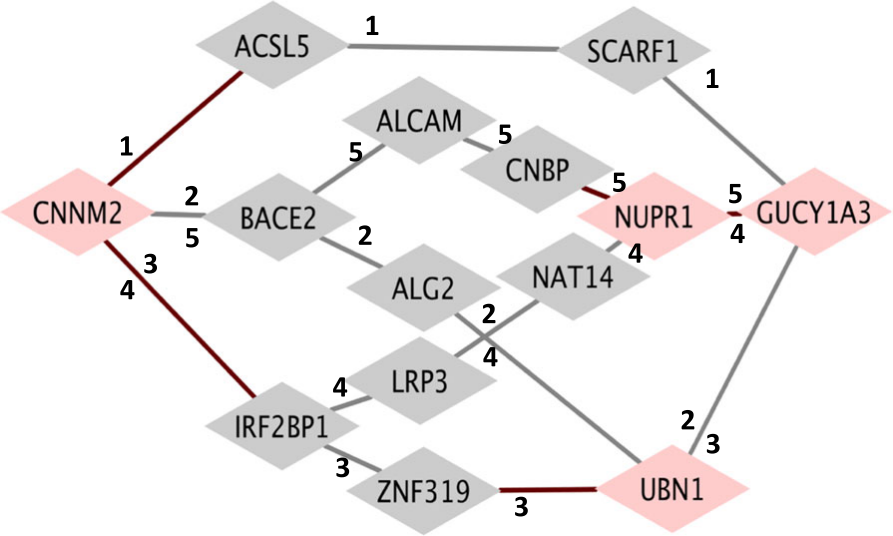
Shortest paths between candidate genes in large-scale co-expression network. Five shortest paths between CNNM2 and GUCY1A3 in co-expression network built using ~13,000 transcripts in artery. Nodes (genes) labeled in pink and edges (pairs) labeled in red are significant in a CATHGEN based logistic model explaining MI by expression of each gene in the path and each pairwise interaction term along the path (i.e. logit(MI) ~ RNA1 + RNA2 + RNA3 + RNA1* RNA2 + RNA2 * RNA3 + additional covariates)

Underlying physiologic mechanisms linking gene expression and MI are indirectly reflected in the sparse networks. To overcome the limitations of these sparse networks, we built large-scale co-expression networks around the 41 candidate genes using 13,000 additional transcripts. At this scale, we expect to observe more biologically credible connections than in a small co-expression network limited to candidate genes. None of the candidate genes were directly co-expressed in these larger networks, indicating that these represent biological processes that are co-regulated but not necessarily in the same pathway. Our analysis revealed indirect connections, i.e., ‘relay’ transcripts that serve to connect candidate genes. Figure 5 displays the shortest paths in blood between a robust pair of candidate mRNAs that interacts in the context of disease: CNNM2|GUCY1A3.

We evaluate the disease relevance of these intermediary co-expressions by considering a logistic model in CATHGEN with all individual RNAs and pairwise non-linear interactions defined by this path in the large-scale network. In the five shortest paths between CNNM2 and GUCY1A3, we find four individual genes (CNNM2, NUPR1, UBN1, GUCY1A3), and five interactions (CNNM2|ACSL5, CNNM2|IRF2BP1, ZNF319|UBN1, CNBP|NUPR1, NUPR1|GUCY1A3) associated with MI. These links between candidate genes serve as additional candidate genes and interactions.

### Testing for epistasis in non-linear interacting candidate gene pairs

Having identified pairwise interactions significant for MI on the RNA level, we asked whether similar interactions occur between SNPs in candidate genes (pairwise epistasis). Using the Framingham Heart Study, we applied the same approach of resampled conditional logistic regression used for RNA interactions. For the 16 candidate genes that were either differentially expressed or formed an interacting pair in the Framingham Heart Study, we considered all possible pairwise interactions between candidate SNPs (GWAS hits published in the GWAS catalog and eQTLs published by GTEx) in the 16 genes. This approach yielded several examples with evidence of epistasis (Additional file 8), but no evidence for an epistatic interaction between variants in those pairs of genes that exhibited an interaction on the RNA level. Non-linear interactions at the genotype level may be less robust than those observed at the level of RNA expression which integrates both genetic and non-genetic factors.

### MI candidate genes also associated with hypercholesterolemia

We performed an identical analysis of single RNAs and RNA pairs in CATHGEN using a related trait, hypercholesterolemia (defined as previous diagnosis and/or treatment of hypercholesterolemia by a physician). Six individual genes (MIA3, CNNM2, HDAC9, LDLR, PLG, TRIB1) and 146 gene pairs with significant interaction terms in the logistic model were associated with hypercholesterolemia (Additional files 9 and 10). The number of MI-based candidate genes differentially expressed in hypercholesterolemia is lower than in MI, and most genes identified for the two traits are different. Three individual genes (MIA3, CNNM2, and TRIB1) and 44 gene pairs overlap between MI and hypercholesterolemia (Additional files 3 and 10). Given hypercholesterolemia is a risk factor for MI this expected overlap suggests that these individual genes and gene pairs relate to MI through lipid metabolism.

## Discussion

The goal of this study was to find mRNA expression patterns (individual mRNAs, non-linear interactions, and paths in large-scale networks) associated with myocardial infarction. Comparison of mRNA profiles in blood from healthy controls and MI cases served to identify 14 differentially expressed candidate genes and 153 non-linear interactions, while co-expression networks in blood and artery from healthy individuals in GTEx supported 31 of these interactions and brought in additional RNAs connecting potential candidate genes. The results support a role for non-additive dynamic interactions between candidate genes in MI.

### mRNA expression guides interpretation of GWAS-based candidate genes

As an intermediate phenotype, gene expression can guide interpretation of GWAS findings [25, 26]. Focusing on mRNA expression of GWAS based candidate genes, we identified 14 individual RNAs differentially expressed in MI. FES, FURIN, PHACTR1, IL6R, RAI1, and UBE2Z were significant in both CATHGEN and FHS while PDGFD, TRIB1, RAI1, LDLR, GGCX, and SLC22A4 had been identified previously in the literature as differentially expressed (Additional file 11). The same approach applied to hypercholesterolemia identified three genes also associated with MI: MIA3, CNNM2, and TRIB1; suggesting their role in MI could be mediated through lipid metabolism. In a study of expression profiles in LCLs and B cells treated with statins, TRIB1 along with seven other genes (LDLR, WDR12, LIPA, EDNRA, TCF21, GUCY1A3, PDGFD), differentially expressed only in MI in our study, also exhibit differential expression upon statin exposure (*p* < 0.05), and therefore, may play a role in response to statin therapy [27].

### Pairs of mRNAs contain information relevant to MI and are biologically plausible

Previous analyses of differential expression have focused on individual genes as the basis for assigning pathways or networks. With the hypothesis that coronary artery disease results from disrupted interactions between gene products, we considered pairwise non-linear interactions between candidate mRNAs. We identified multiple pairs with interaction terms associated with MI, two of which replicated across cohorts: CNNM2|GUCY1A3 and CNNM2|ZEB2. Disease related changes of the dynamic interactions between these mRNA pairs identifies robust odds ratios for MI. Supporting a role of non-linear interactions in logistic models of disease risk, the log odds is not a monotone function of mRNA expression. Shown in Fig. 2, when expression of GUCY1A3 is high, increasing expression of CNNM2 associates with risk, whereas when expression of GUCY1A3 is low, increasing expression of CNNM2 is protective, potentially with high predictive power. Therefore, dynamic interaction terms reveal otherwise hidden higher-order relationships between candidate genes. This emphasizes the potential utility of pairwise interactions between mRNA expression profiles as a biomarker for disease.

Since in addition to the non-linear interaction, we find CNNM2 and GUCY1A3 co-expressed in small-scale networks in both artery and blood, the question arises about the underlying mechanism that regulates these genes, the disruption of which may lead to MI. A physiologic connection between CNNM2 and GUCY1A3 indeed reveals disease relevant processes. A Mg^2+^ transporter, CNNM2 interacts in the context of MI with GUCY1A3, a Mg^2+^ dependent guanylate cyclase responsive to nitric oxide. Mutations in CNNM2 have been implicated in familial hypomagnesemia with symptoms including cardiac arrhythmias [28], while common variants of uncertain functions in GUCY1A3 have been implicated in hypertension by GWAS, and rare non-synonymous variants in disorders of vascular tone and myocardial infarction [29–34]. Furthermore, transient hypomagnesemia has been reported in acute MI with vasospasm as a proposed mechanism [35]. We suggest imbalanced mRNA expression of CNNM2 predisposes individuals to hypo- or hypermagnesemia, which could magnify the effect of genetic variants altering GUCY1A3 expression, either directly or *via* intermediate mRNAs, to generate conditions that favor MI.

The relationship between these two genes and Mg^2+^ is further maintained in one of the shortest paths between the two in a larger co-expression network. The path CNNM2|ACSL5|SCARF1|GUCY1A3 appears to be united by the common themes of magnesium and lipid processing. ACSL5 binds magnesium ions and activates long-chain fatty acids, while SCARF1 acts as a scavenger receptor to regulate uptake of modified LDL – levels of which are decreased in the presence of magnesium [36]. In addition to observing an association between CNNM2 and GUCY1A3 individually with MI, the interaction term between CNNM2 and ACSL5 is also significant (*p* < 0.05) indicating that relationships determined by co-expression patterns in healthy individuals can be informative of disease.

Co-expression in healthy individuals of the same pair of genes that exhibit a non-linear interaction associated with disease supports the biological plausibility of the pair. In this study, 20 % of RNA pairs with non-linear interactions associated with disease are also co-expressed in healthy subjects, indicating co-expression may serve as an additional criterion to prioritize significant interactions. We have identified disease-associated dynamic interactions between mRNA transcripts of strong candidate genes, gleaned from expression profiles in whole blood and recapitulated by co-expression in artery. The results support the notion that RNA profiles in blood can reveal disease-relevant processes in the tissue of interest.

### Evidence for disrupted network structure in MI

Non-linear pairwise interactions associated with MI form a well-connected graph that includes virtually all candidate genes, suggesting that relevant information about disease is dispersed across a broad network. We propose that there is a fundamental difference in detecting genes involved in MI pathophysiology by considering an additive *versus* a non-linear effect. Defining pairs based on an additive (rather than interactive) model reduces the number of genes included in the network by half, whereas non-linear interactions appear better to capture disease risk and may prove useful in accounting for genetic factors related to complex diseases.

## Conclusions

Considering pairwise interactions between candidate genes reveals strong, disease-relevant pairs – a possible entry point for broad study of MI systems biology. Our results further demonstrate that mRNA expression profiles encoded by a limited number of candidate genes yields sparse interacting networks. Serving as an anchor to extend the analysis genome-wide, we then searched for relay genes in larger networks, confirming additional candidate genes and identifying novel ones. Additional work is needed to elucidate higher order interactions and further assess potential utility of the dynamic interactions observed here in clinical biomarker panels.

## Methods

### Data

#### CATHeritization GENetics (CATHGEN)

Expression data, genotypes, and clinical phenotypes were acquired from CATHeritization GENetics (CATHGEN) *via* dbGaP Project #5358 (dbGaP accession number phs0000703 on 25 March 2015). Expression levels had been determined using Illumina HumanHT-12 v3 in RNA from whole blood at the time of catheterization and recruitment to the study. We considered age (phv00197199), gender (phv00197207), and race (phv00197206) as additional covariates in our models recorded in pht003672. Myocardial infarction (MI), defined by a previous recorded history of MI (phv00197212) or a non-zero CAD index (phv00197202) recorded in pht003672, was used as a phenotype/outcome variable. CAD index was used in addition to previous history of MI to define cases because of a large number of missing values regarding MI history. Based on work by both Kitsios et al. and Nikpay et al., we anticipate similar results for cases defined solely by MI status and those defined by CAD index [37–39]. Hypercholesterolemia (phv00197204) recorded in pht003672 was also used as a phenotype/outcome variable. Our access of this study was approved by the Ohio State University IRB (Protocol #2013H0096).

#### Framingham Heart Study (FHS)

Expression data, genotypes, and clinical phenotypes were acquired from the Framingham Heart Study (FHS) *via* dbGaP Project #5358 (dbGaP accession number phs000007 on 21 July 2014). Expression levels had been determined using the Affymetrix Human Exon 1.0 ST Array in RNA from whole blood. Expression measures were not in anyway timed relative MI; however virtually all MI events preceded measurement of gene expression – at times by several decades (Additional file 12). Genotypes were taken from the SHARe substudy that used the OMNI5M genotyping array. We considered age (phv00177930), gender (phv00177929), and family assignment (phv00024067) as covariates in our models recorded in tables pht003099 and pht000183. History of MI, defined by one of the following recorded events: 1 = MI recognized, with diagnostic ECG, 2 = MI recognized, without diagnostic ECG, with enzymes and history, 3 = MI recognized, without diagnostic ECG, with autopsy evidence, new event (see also code 9), 4 = MI unrecognized, silent, 5 = MI unrecognized, not silent, or 8 = Questionable MI at exam 1 (variable phv00036469 recorded in table pht000309), was used as the phenotype/outcome variable. Our access of this study was approved by the Ohio State University IRB (Protocol #2013H0096).

#### Genotype and Tissue Expression Project (GTEx)

Tissue specific RNAsequencing data was acquired from the Genotype and Tissue Expression Project (GTEx) *via* dbGaP Project #5358 (dbGaP accession number phs000424 in April 2014). RNAsequencing had been generated using poly-adenylated priming with reads aligned to HG19, Gencodev12. For further details see Lonsdale et al. and the GTEx website (http://www.gtexportal.org/home/documentationPage) [40]. We considered tibial artery tissue (137 samples) and whole blood (190 samples). We used tibial artery instead of coronary artery for a target tissue for MI pathophysiology, because the number of coronary artery tissue samples in GTEx was insufficient at the time of this analysis. As plaque formation also occurs in peripheral arteries, tibial artery is also suitable for this study. The entire artery sample was used with no isolation of endothelial *versus* smooth muscle cells, etc. We used the number of reads aligned to a given transcript as the measure of expression. In each tissue, we selected only those transcripts that were non-zero in more than 65 % of samples. Accordingly, in the small networks, we analyzed 132 transcripts in artery and whole blood; while in the large networks, we analyzed 8080 transcripts in artery, and 6705 in whole blood.

The majority of individuals in CATHGEN, FHS, and GTEx are Caucasian. Accordingly, our results are not generalizable to more diverse populations.

### Gene list

Candidate genes for differential expression analysis and small co-expression networks were selected from the CAD Genome Wide Association Study performed by the CARDIOGRAMplusC4D consortium published in 2014 [1]. Forty-one of the candidate genes assigned to these loci had corresponding probes on expression arrays used in both CATHGEN and the Framingham Heart Study. All probes corresponding to these genes were tested for an association between mRNA expression and MI.

For the large co-expression networks, we considered transcripts included on the Illumina HumanHT-12_V4 expression array and added any candidate genes identified through literature review and use of large databases (e.g. dbGaP, OMIM) that were not already included. We analyzed a total of 12,913 transcripts in 4098 genes. We obtained transcripts for each gene with the aid of ‘biomaRt’ package in the statistical language R (cran.r-project.org).

### Association of gene expression and myocardial infarction

#### CATHGEN: logistic regression

Association between expression and MI (or hypercholesterolemia) was assessed using logistic regression. We considered age, race, and gender as additional covariates in the model (*n* = 1250; 359 cases and 891 controls). Expression was considered significant with a *p*-value < 0.05. Logistic regression was performed considering expression of a single gene, pairs of genes (with and without an interaction term), and paths derived from tissue-specific co-expression networks. The explicit models are outlined below. Computations were done using the function ‘glm’ in the ‘stats’ package in R.

#### Framingham Heart Study: bootstrapped conditional logistic regression

Association between expression and MI was assessed using conditional logistic regression (CLR) in R as implemented in package ‘survival’. One hundred and ninety-three cases of MI were matched to 4084 controls on age (+/− 2 years), gender, and different family assignment (note: all individuals in FHS are Caucasian). CLR was then performed 5000 times, each time using all 193 cases but with different subsets (resamples) of the 4084 matched controls. For each CLR associated with an individual case–control pairing, expression of a given gene was considered significant if the null hypothesis (effect-size of gene expression on MI status is zero) was rejected at *p* < 0.05 by the Wald test. For each effect (main effect as well as interaction effect) in the logistic model, the percent of CLR resamples where expression was significantly associated with MI was reported. CLR considering expression of a single gene and pairs of genes (with and without an interaction term) was performed. The estimation of effects of individual variants and pairs of variants acting in epistasis was performed the same way as in the case of expression data. The explicit models are outlined below.

##### Expression of individual genes

CATHGEN: MI ~ gene expression + age + race + gender

FHS: MI ~ gene expression

##### Expression of pairs of genes

CATHGEN: MI ~ gene A expression + gene B expression + age + race + gender

MI ~ gene A expression + gene B expression + gene A expression * gene B expression + age + race + gender

FHS: MI ~ gene A expression + gene B expression

MI ~ gene A expression + gene B expression + gene A expression * gene B expression

##### Expression of paths (CATHGEN only)

CATHGEN: MI ~ gene A expression + gene 1 expression + gene 2 expression … gene n expression + gene B expression + gene A expression*gene 1 expression + gene 1 expression*gene 2 expression … + gene n expression*gene B expression + age + race + gender

Note: In FHS covariates (age, gender, and family) were considered by using matched case–control design.

### Estimating odds of myocardial infarction for interacting gene pairs

We used the confidence bounds generated by the bootstrapped CLR in FHS to calculate the odds ratio of MI given that the expression of gene A is at its mean and increases by one standard deviation as a function of expression of gene B.

$$ odds\left( Ex\_A+sd\left( Ex\_A\right)\right)/ odds\left( Ex\_A\right) $$

where *Ex_A* and *sd(Ex_A)* denote the expression level and the standard deviation of expression of gene A respectively.

### Co-expression network procedure

#### Network construction procedure

We evaluated the strength and robustness of co-expression patterns in a Gibbs-Boltzmann model using Renyi divergence, with a free parameter α, as described in detail in Additional file 4 [41]. A similar approach, called ARACNE [23], has been successfully used in a wide variety of applications [42]. Briefly, our algorithm may be described as follows: Suppose that the set of m transcripts and tissue are fixed. We consider k = 50000 resamples of I individuals for the small networks and k = 300 resamples of I individuals for the large networks. For each random subset of individuals, a weighted, undirected graph of m nodes (m being the number of transcripts) is generated. Edge weights are defined by calculating the pairwise Renyi mutual information and edges are pruned using the Data Processing Inequality (as described in the Additional file 4). Next with each random subset of individuals we associated an m x m adjacency matrix and based on k resamples generate the m x m average adjacency matrix (‘consensus matrix’) [M(i,j)]. Each (i,j)-entry is the proportion of resamples where a particular co-expression of a pair of transcripts (i,j) was observed.

The analysis was implemented in the statistical software R (cran.r-project.org), in particular the package ‘infotheo’ for discretizing the data (we use the method ‘equalfrequencies’) and the package ‘minet’ with function ‘aracne’ for pruning indirect interactions.

#### Preserved co-expressions

We focused on the co-expressions that have a sample frequency above 0.5 due to the following observation. Consider a direct interaction between two RNAs *X*_*i*_ and *X*_*j*_ and assume that there exists a triangle (*X*_*i*_, *X*_*j*_, *X*_*l*_) with an indirect interaction (say between *X*_*i*_ and *X*_*l*_). If the true values of the Renyi mutual information for (*X*_*i,*_*X*_*j*_) and for (*X*_*i*_, *X*_*l*_) are arbitrarily close (but *D*_*α*_ (*X*_*i*_, *X*_*j*_) > *D*_*α*_ (*X*_*i*_, *X*_*l*_)), then the sample estimate of *D*_*α*_ (*X*_*i*_, *X*_*j*_) should be greater than the sample estimate of *D*_*α*_ (*X*_*i*_, *X*_*l*_) at least half of the times. In other words, since a direct interaction between *X*_*i*_ and *X*_*j*_, is stronger than their indirect interaction (acting *via* an intermediate RNA), then the co-expression of (*X*_*i*_, *X*_*j*_) is expected to appear in at least half of resamples in the consensus matrix.

## Additional files

Additional file 1: GWAS-based candidate genes. The CardioGRAMC4D Consortium reported 50 loci implicated in risk of CAD and assigned these variants to one or more genes in the locus. Included is a summary table of the variants and assigned genes. Also included is any evidence identified by GTEx for an eQTL associated with expression of the assigned genes and the linkage structure between these eQTLs and the reported GWAS variants. (XLSX 47 kb) Additional file 2: Distribution of *p*-values. Histograms of *p*-values for 5000 bootstrap replicates of conditional logistic regression (MI ~ gene expression) in the Framingham Heart Study for those genes determined as significant in CATHGEN that did not meet the criteria for being associated with MI in FHS. FURIN, IL6R, RAI1, and UBE2Z were classified as informative of MI based on right-skewed distribution and are shown in Fig. 1. CNNM2, GUCY1A3, MRAS, MIA3, PDGFD, PEMT, and LPAL2 did not show evidence of differential expression given almost uniformly distributed *p*-values and are shown here. (PDF 188 kb) Additional file 3: Non-linear interacting pairs in CATHGEN. Included are non-linear interacting pairs identified in CATHGEN for significant (*p* < 0.05) interaction term in regression model. Probe IDs, gene names, and ENSGs are reported along with *p*-value for interaction term and proportion of resamples the gene pair was co-expressed in tissue specific co-expression networks. (XLSX 75 kb) Additional file 4: Introduction of the network construction algorithm. Detailed background and introduction of the network construction algorithm presented. (DOCX 115 kb) Additional file 5: Overview of co-expression network construction and resampling procedure. Tissue specific co-expression networks were built based on pairwise mutual information values between RNA transcripts measured by Renyi divergence. For each tissue type, multiple networks were generated using different random sub-samples of individuals. Networks were pruned based on the Data Processing Inequality and a consensus was taken from the resulting matrices. We report the resample frequency (i.e. proportion of networks with observed co-expression) as a measure of the robustness of co-expression. (PDF 214 kb) Additional file 6: Tissue specific co-expressions. Gene pairs co-expressed in artery, blood, and both artery and blood. Reported as ENSGs with proportion of resamples in co-expression network. (XLSX 47 kb) Additional file 7: Co-expression as a filter for disease-relevant interactions. Proportion of true null hypotheses (estimated *via* the qvalue package in R) when testing differential expression of: single genes, gene pairs, co-expressed pairs. A lower proportion of the true null indicates greater specificity of the model. (PDF 119 kb) Additional file 8: Epistatic pairs. Considering interaction terms between candidate SNPs (GWAS hits or eQTLs) for all combinations of genes with expression patterns associated either individually or in combination with MI in the Framingham Heart Study, we identify five instances of epistasis. Resampled conditional regression was performed considering the main effect of each SNP and an interaction term. Reported is the percent of significant (*p* < 0.05) resamples out of 5000 for each term. (DOCX 155 kb) Additional file 9: Differentially expressed candidate genes in hypercholesterolemia. *P*-values of association between expression of probe ID (labeled by assigned gene) and MI (A) or hypercholesterolemia (B) measured in CATHGEN using logistic regression with age, race, and gender as additional covariates. C. Venn diagram displaying overlap between genes individually significant in MI and hypercholesterolemia. (PDF 1435 kb) Additional file 10: Differentially expressed candidate gene pairs in hypercholesterolemia. Connected graph formed by pairs of genes with a significant interaction term in CATHGEN using a logistic model accounting for MI (A) or hypercholesterolemia (B) with expression of gene 1, expression of gene 2, expression of gene 1 * expression of gene 2, and additional covariates (age, race, gender). C. Intersection of graphs for MI and hypercholesterolemia. Pink nodes indicate genes that are individually significant in MI (see Additional file 9) and orange nodes indicate genes that are individually significant in hypercholesterolemia (see Additional file 9). (PDF 1681 kb) Additional file 11: Replication of differentially expressed genes. A. Venn diagram displaying number of differentially expressed genes identified by previous studies reporting differentially expressed genes in MI/CAD. B. Venn diagram displaying overlap of differentially expressed genes between CATHGEN and FHS cohorts as determined by our analysis. C. Venn diagram displaying overlap of candidate genes considered in our analysis and those previously identified as differentially expressed in the literature. Gene names colored in red were identified by our analysis as differentially expressed. Note: gene names reported by each study were first converted to ENSG identifiers in order to ensure they were directly comparable. Gene names that did not map to an ENSG were not included. (PDF 288 kb) Additional file 12: Timing of MI relative to measure of gene expression in FHS. (DOCX 49 kb)

## Abbreviations

CAD: Coronary artery disease
CATHGEN: CATHeterization in GENetics
FHS: Framingham heart study
MI: Myocardial infarction
