# Erratum to: Non-linear interactions between candidate genes of myocardial infarction revealed in mRNA expression profiles

**DOI:** 10.1186/s12864-016-3349-z

**Published:** 2016-12-02

**Authors:** Katherine Hartmann, Michał Seweryn, Samuel K. Handelman, Grzegorz A. Rempała, Wolfgang Sadee

**Affiliations:** 1 College of Medicine Center for Pharmacogenomics, The Ohio State University Wexner Medical Center, Biomedical Research Tower, 460 W 12th Avenue, Columbus, OH USA; 2 Department of Molecular Virology, Immunology, and Medical Genetics, The Ohio State University, Biomedical Research Tower, 460W 12th Avenue, Columbus, OH USA; 3 Faculty of Mathematics and Computer Science, University of Łodz, Łodz, Poland; 4 Division of Biostatistics, College of Public Health, The Ohio State University, 250 Cunz Hall, 1841 Neil Avenue, Columbus, OH USA; 5 Mathematical Biosciences Institute, The Ohio State University, Jennings Hall 3rd Floor, 1735 Neil Avenue, Columbus, OH USA

## Erratum

In the original publication of this article [1], “Samuel K. Handleman” should have been listed as “Samuel K. Handelman”.
