# Supplementary figures and images for: Non-linear interactions between candidate genes of myocardial infarction revealed in mRNA expression profiles
Source: BMC Genomics. 2016 Sep 17;17:738. doi: 10.1186/s12864-016-3075-6 (PMC5027110; doi:10.1186/s12864-016-3075-6)

***CNNM2***

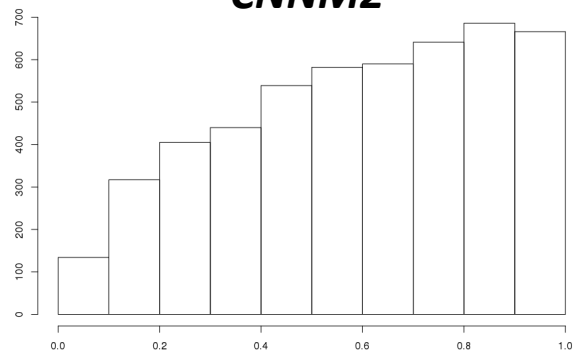

***GUCY1A3***

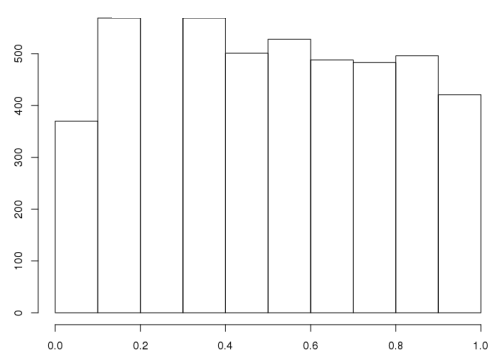

***MRAS***

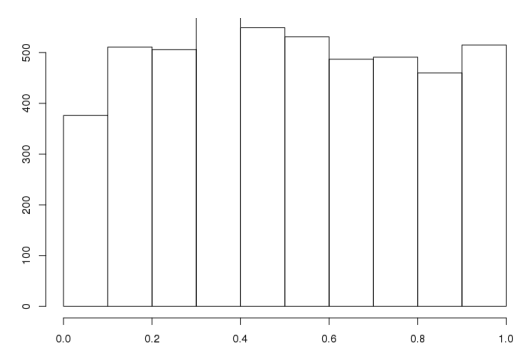

***MIA3 #1***

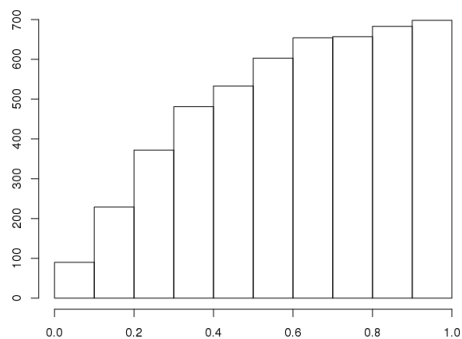

***MIA3 #2***

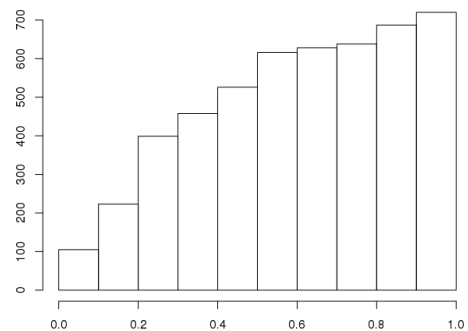

***PDGFD***

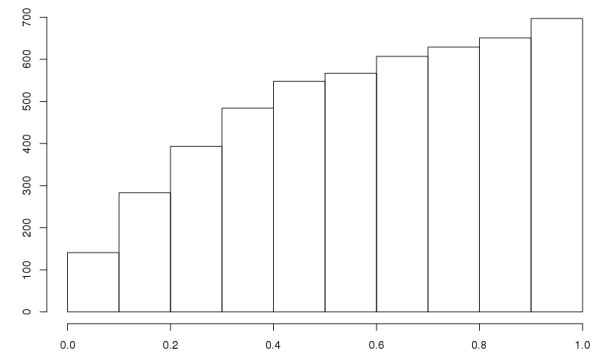

***PEMT***

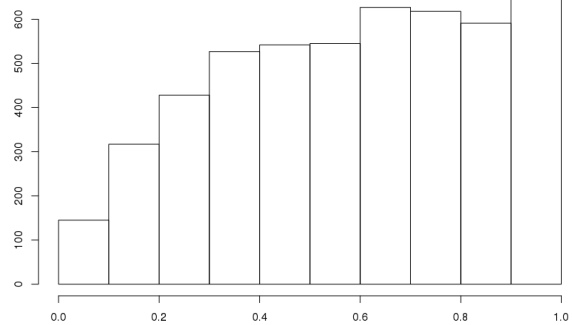

***LPAL2***

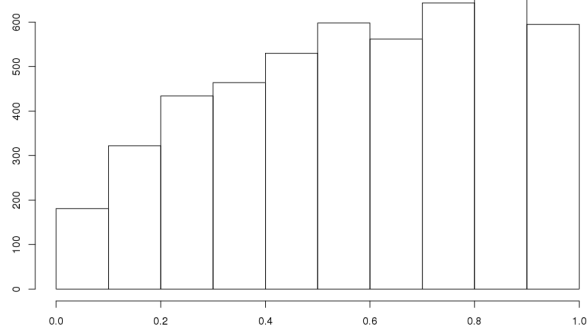

Supplement: Additional file 2: — Distribution of p-values. Histograms of p-values for 5000 bootstrap replicates of conditional logistic regression (MI ~ gene expression) in the Framingham Heart Study for those genes determined as significant in CATHGEN that did not meet the criteria for being associated with MI in FHS. FURIN, IL6R, RAI1, and UBE2Z were classified as informative of MI based on right-skewed distribution and are shown in Fig. 1. CNNM2, GUCY1A3, MRAS, MIA3, PDGFD, PEMT, and LPAL2 did not show evidence of differential expression given almost uniformly distributed p-values and are shown here. (PDF 188 kb) [file 12864_2016_3075_MOESM2_ESM.pdf]

## Co-expression of genes A, B, and C in tissue x:

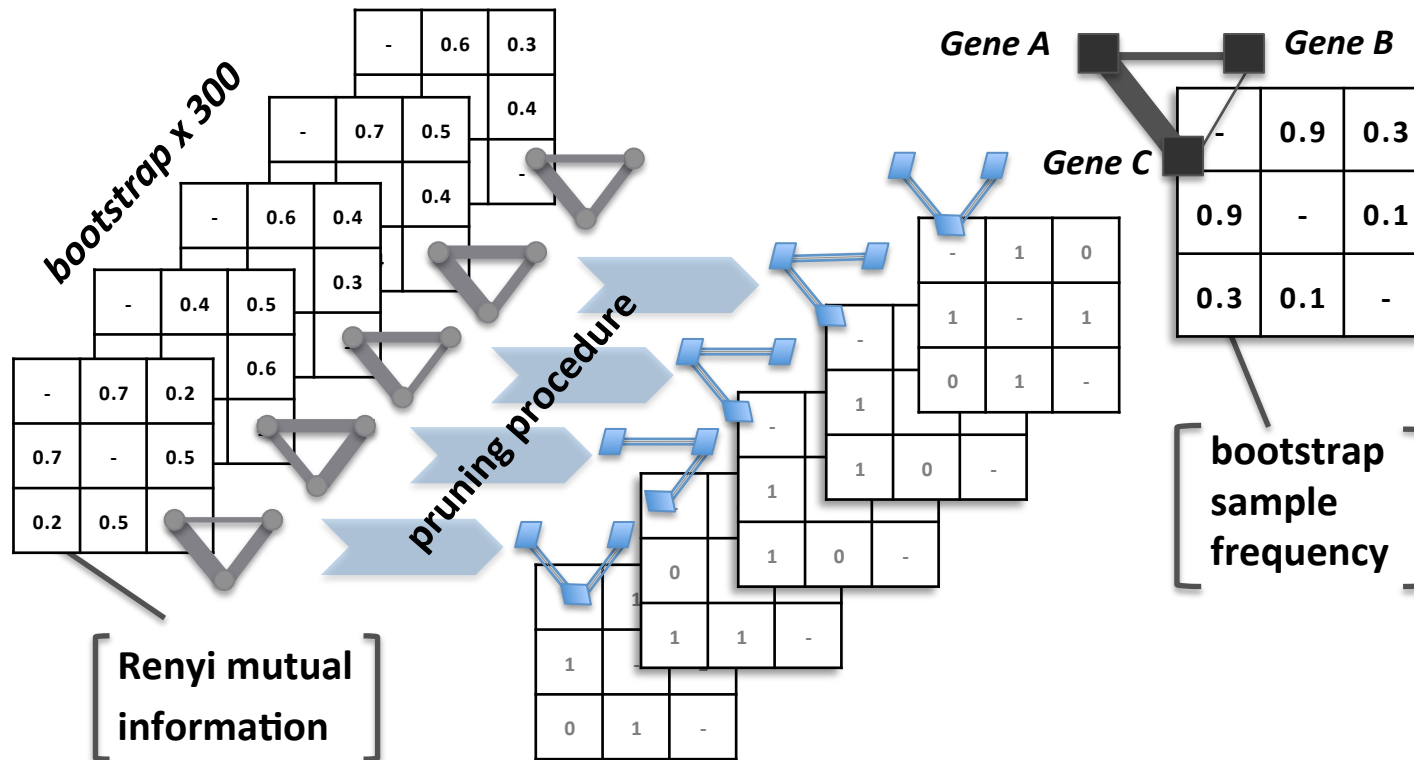

Supplement: Additional file 5: — Overview of co-expression network construction and resampling procedure. Tissue specific co-expression networks were built based on pairwise mutual information values between RNA transcripts measured by Renyi divergence. For each tissue type, multiple networks were generated using different random sub-samples of individuals. Networks were pruned based on the Data Processing Inequality and a consensus was taken from the resulting matrices. We report the resample frequency (i.e. proportion of networks with observed co-expression) as a measure of the robustness of co-expression. (PDF 214 kb) [file 12864_2016_3075_MOESM5_ESM.pdf]

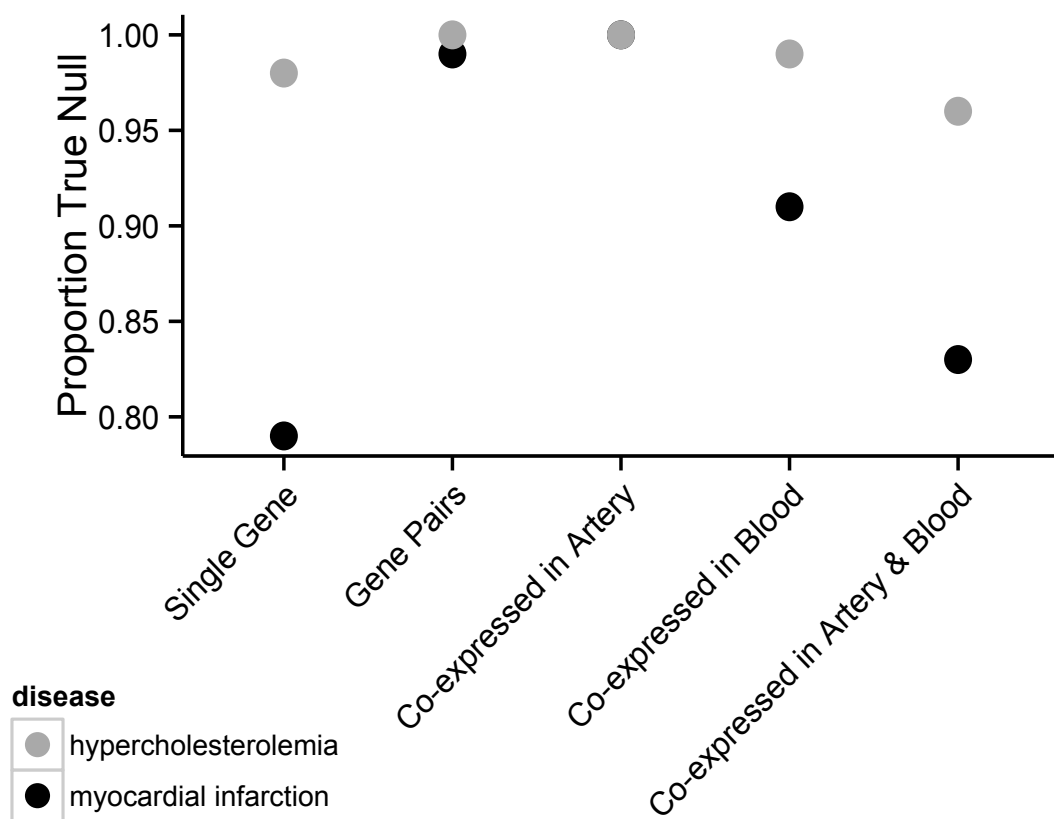

Supplement: Additional file 7: — Co-expression as a filter for disease-relevant interactions. Proportion of true null hypotheses (estimated via the qvalue package in R) when testing differential expression of: single genes, gene pairs, co-expressed pairs. A lower proportion of the true null indicates greater specificity of the model. (PDF 119 kb) [file 12864_2016_3075_MOESM7_ESM.pdf]

## A CATHGEN Myocardial Infarction

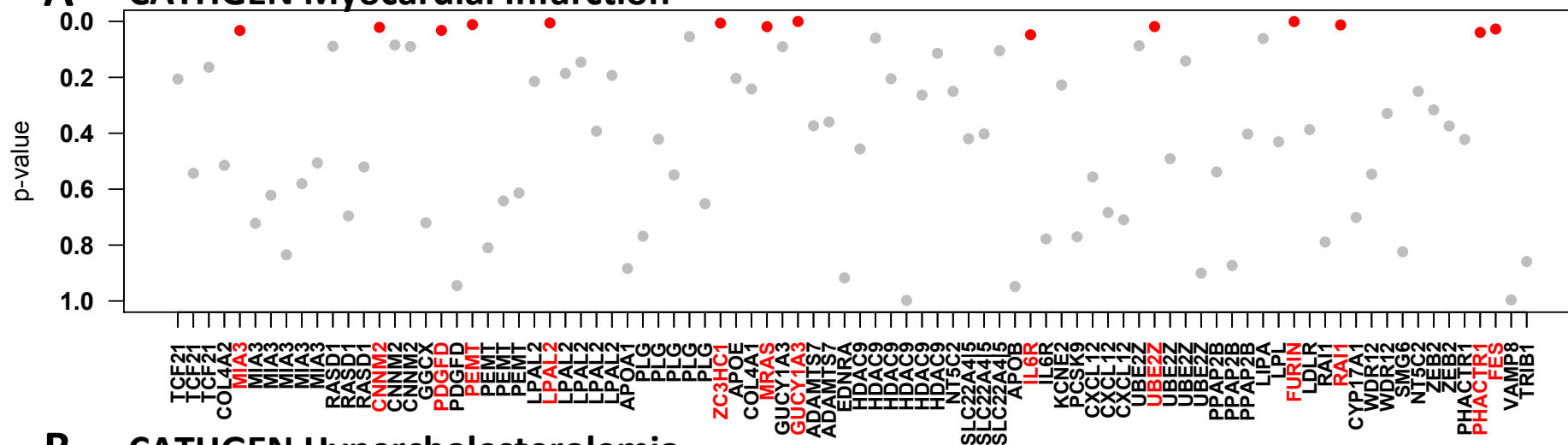

## B CATHGEN Hypercholesterolemia

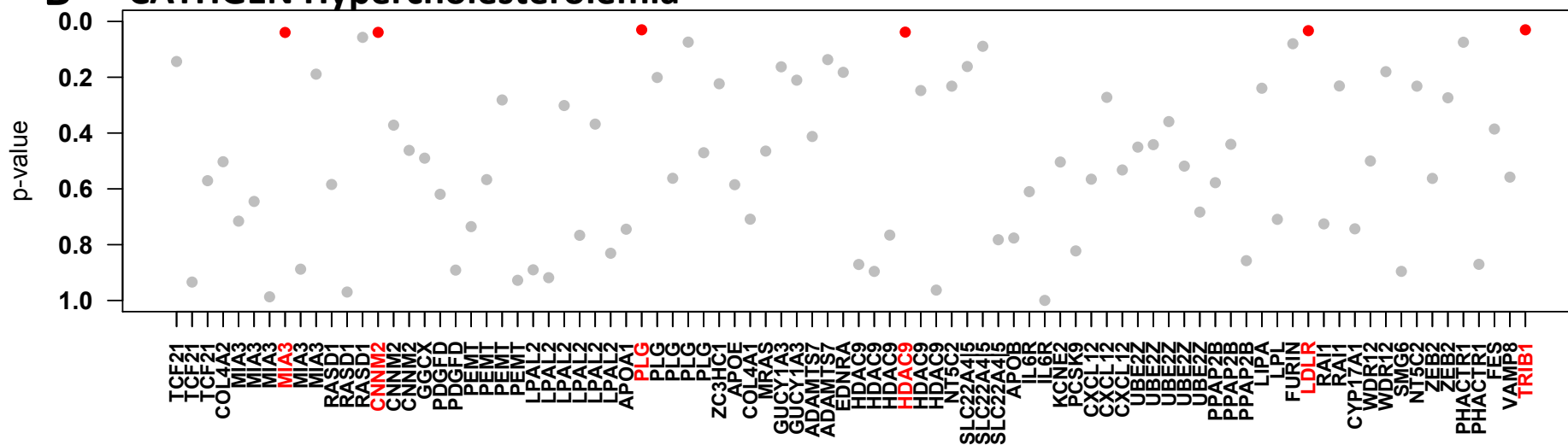

## C

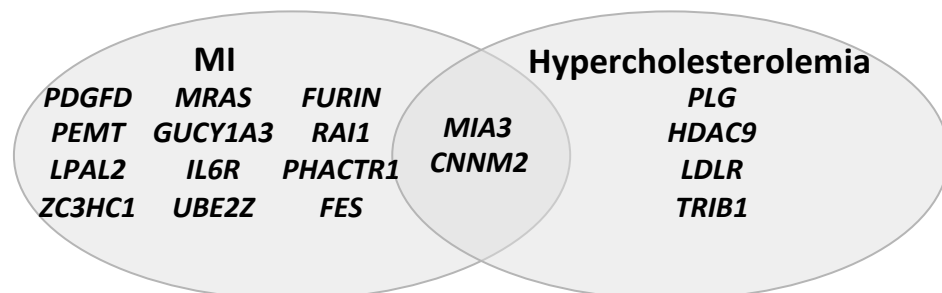

Supplement: Additional file 9: — Differentially expressed candidate genes in hypercholesterolemia. P-values of association between expression of probe ID (labeled by assigned gene) and MI (A) or hypercholesterolemia (B) measured in CATHGEN using logistic regression with age, race, and gender as additional covariates. C. Venn diagram displaying overlap between genes individually significant in MI and hypercholesterolemia. (PDF 1435 kb) [file 12864_2016_3075_MOESM9_ESM.pdf]

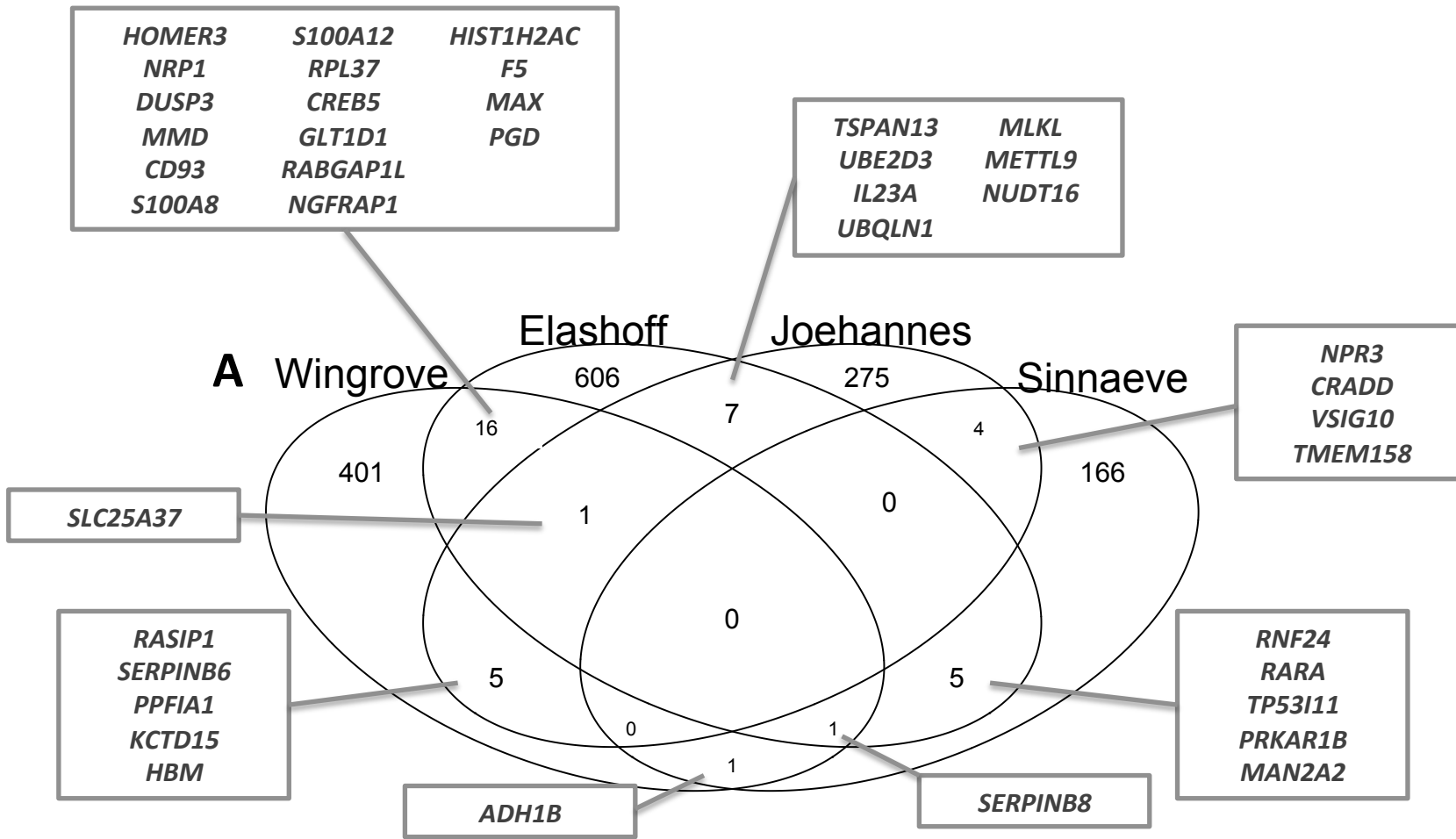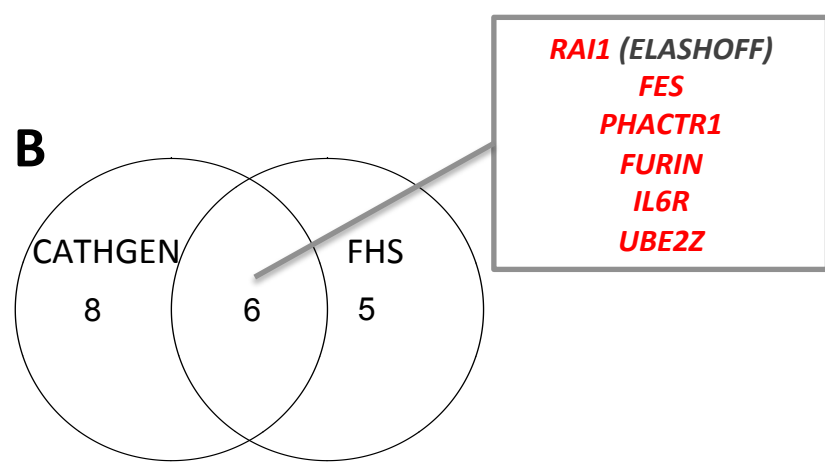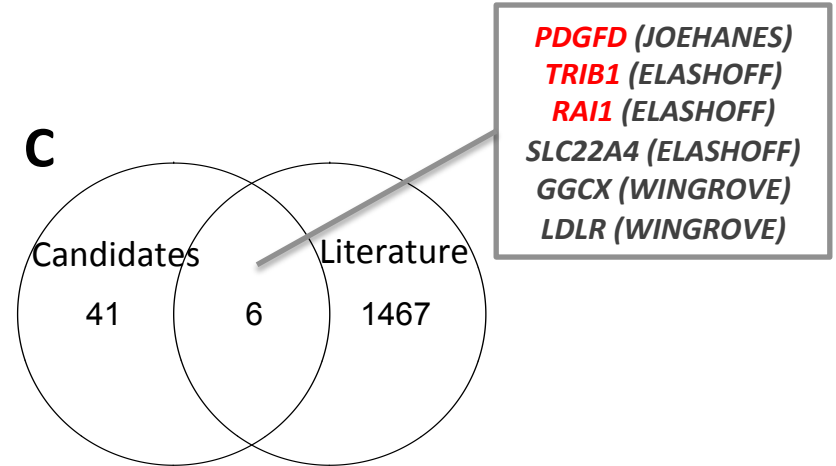

Supplement: Additional file 11: — Replication of differentially expressed genes. A. Venn diagram displaying number of differentially expressed genes identified by previous studies reporting differentially expressed genes in MI/CAD. B. Venn diagram displaying overlap of differentially expressed genes between CATHGEN and FHS cohorts as determined by our analysis. C. Venn diagram displaying overlap of candidate genes considered in our analysis and those previously identified as differentially expressed in the literature. Gene names colored in red were identified by our analysis as differentially expressed. Note: gene names reported by each study were first converted to ENSG identifiers in order to ensure they were directly comparable. Gene names that did not map to an ENSG were not included. (PDF 288 kb) [file 12864_2016_3075_MOESM11_ESM.pdf]
