# Supplementary material for: Non-linear interactions between candidate genes of myocardial infarction revealed in mRNA expression profiles
Source: BMC Genomics. 2016 Sep 17;17:738. doi: 10.1186/s12864-016-3075-6 (PMC5027110; doi:10.1186/s12864-016-3075-6)
Supplement: Additional file 4: — Introduction of the network construction algorithm. Detailed background and introduction of the network construction algorithm presented. (DOCX 115 kb) [file 12864_2016_3075_MOESM4_ESM.docx]

**Additional file 4: Supplementary Methods: Introduction of network construction algorithm**

*Model*

We assume that the joint probability mass function of the expression of m transcripts $\left( X_{1,}\ldots,X_{m} \right)$ may be represented by individual expression levels $X_{i},i=1,2,\ldots,m$ and pairwise interactions (co-expressions) $\left( X_{i},X_{j} \right),i\neq j,i,j=1,2,\ldots,$. This is usually stated in terms of the Gibbs measure [1], or the Hamiltonian of the system, as follows:

$$P\left( X_{1}=x_{1,}\ldots,X_{m}=x_{m} \right)=\frac{1}{Z}exp\left( -\sum\phi_{i}\left( x_{i} \right)-\sum\phi_{ij}\left( x_{i},x_{j} \right) \right)$$

where Z is the normalization constant and $\phi_{i},\phi_{ij},i\neq j,i,j=1,2,\ldots,m$ are potential functions. Such assumptions have been exploited in many methods for building genetic co-expression networks [2] and also in the theory of Markov random fields [1].

*Measure of co-expression*

To quantify co-expression of transcripts we use the generalized one parameter family of Renyi divergence measures [3]:

$D_{\alpha}\left( p\parallel q \right)=\frac{1}{\alpha-1}ln\left( \sum p^{\alpha}q^{1-\alpha} \right)$ where $p=\left( p_{1,}\ldots,p_{k} \right),q=\left( q_{1,}\ldots,q_{k} \right)$

are probability vectors. For discrete distributions, the Renyi divergence shares a wide variety of properties with the standard Kullback-Leibler divergence [4] and the chi-square distance. We quantify the strength of pairwise interactions (RNA co-expression) by measuring the ‘distance’ (Renyi divergence) between the (marginalized) bivariate distribution of $\left( X_{i},X_{j} \right),i\neq j,i,j=1,\ldots,m$ and the product of its marginals:

$$D_{\alpha}\left( X_{i},X_{j} \right):=D_{\alpha}\left( P_{X_{i},X_{j}}\parallel P_{X_{i}}P_{X_{j}} \right)=\frac{1}{\alpha-1}ln\left( \sum{P\left( X_{i}=x_{i},X_{j}=x_{j} \right)}^{\alpha}\left( P\left( X_{i}=x_{i} \right)P\left( X_{j}=x_{j} \right) \right)^{1-\alpha} \right)$$

Following the standard convention for $\alpha=1$, where the Kullback-Leibler divergence between the bivariate distribution and the product of its marginals is called ‘Mutual Information’, we call the above defined functionals ‘Renyi mutual information’.  It is important to note that the Renyi mutual information equals zero if and only if the bivariate distribution equals the product of its marginals (i.e. the expression levels of the two RNAs are independent).

The parameter $\alpha$ in the Renyi mutual information allows for up or down weighting of negative or positive dependencies in the considered two-dimensional contingency table corresponding to the empirical bivariate distribution $\left( X_{i},X_{j} \right)$[5–7]. Here, we aimed to investigate context-dependent co-expression patterns (the context being varying genetic and environmental backgrounds). Therefore, in addition to the resampling procedure (see Additional file 6), we investigated the impact of rare *versu*s frequent bins in the contingency table on co-expression by changing the free parameter $\alpha$. We selected several different values of this parameter (ranging form 0.1 to 3) and observed that the degree distribution of the graph remained unchained whereas we observed a small stability of individual co-expressed pairs. It remains to be determined whether for specific, small scale systems of RNAs a higher (or lower) stability of interactions may be achieved *via* the choice of the $\alpha$ parameter. Nevertheless, we find that the impact of frequent *versus* rare co-expressions may be changed by up- or down-weighting positive and negative dependencies using the free parameter[8].

*Data Processing Inequality*

Note that we assume that the probability mass function may be written in the Gibbs form with only first and second order interactions. In such a setting, Margolin et. al. used the standard Mutual Information as measure of co-expression (estimated via the Gaussian kernel method) and employed the Data Processing Inequality for pruning indirect interactions as part of the ARACNE algorithm for building co-expression networks [2]. We note that the Data Processing Inequality holds for the generalized Renyi divergence measures as well (see also Theorem 1 and Theorem 9 in van Erven & Harremoes, 2014) [9]. Accordingly, we also employ the Data Processing Inequality to prune ‘indirect’ edges from the full graph. Therefore, at each repetition of the resampling procedure our algorithm considers all triangles in the full graph and removes the ‘weakest’ edge (i.e. the co-expression with the lowest value) of each triangle, since, in the simplest Markov-type model, the information represented by the weakest edge in a triangle is explained by the two stronger ones (i.e. the weakest interaction is indirect).

1. Kindermann R, Snell JL: *Markov Random Fields and Their Applications*. *Volume 1*. Providence, Rhode Island: American Mathematical Society; 1980. [*Contemporary Mathematics*]

2. Margolin AA, Nemenman I, Basso K, Wiggins C, Stolovitzky G, Dalla Favera R, Califano A: **ARACNE: an algorithm for the reconstruction of gene regulatory networks in a mammalian cellular context.** *BMC Bioinformatics* 2006, **7 Suppl 1**(Suppl 1):S7.

3. Rényi A: **On Measures of Entropy and Information**. In *Proceedings of the Fourth Berkeley Symposium on Mathematical Statistics and Probability, Volume 1: Contributions to the Theory of Statistics*. The Regents of the University of California; 1961.

4. van Erven T, Harremoes P: **Rényi Divergence and Kullback-Leibler Divergence**. *IEEE Trans Inf Theory* 2014, **60**:3797–3820.

5. Kotz S, Wang Q, Hung K: **Interrelations among various definitions of bivariate positive dependence**. 1990:333–349.

6. Lehmann EL: **Some Concepts of Dependence**. *Ann Math Stat* 1966, **37**:1137–1153.

7. Rempala GA, Seweryn M: **Methods for diversity and overlap analysis in T-cell receptor populations.** *J Math Biol* 2013, **67**:1339–68.

8. Douglas R, Fienberg SE, Lee MLT, Sampson AR, Whitaker LR: **Positive dependence concepts for ordinal contingency tables**. 1990:189–202.

9. van Erven T, Harremoes P: **Rényi Divergence and Kullback-Leibler Divergence**. *IEEE Trans Inf Theory* 2014, **60**:3797–3820.
