# Supplementary material for: Non-linear interactions between candidate genes of myocardial infarction revealed in mRNA expression profiles
Source: BMC Genomics. 2016 Sep 17;17:738. doi: 10.1186/s12864-016-3075-6 (PMC5027110; doi:10.1186/s12864-016-3075-6)
Supplement: Additional file 8: — Epistatic pairs. Considering interaction terms between candidate SNPs (GWAS hits or eQTLs) for all combinations of genes with expression patterns associated either individually or in combination with MI in the Framingham Heart Study, we identify five instances of epistasis. Resampled conditional regression was performed considering the main effect of each SNP and an interaction term. Reported is the percent of significant (p < 0.05) resamples out of 5000 for each term. (DOCX 155 kb) [file 12864_2016_3075_MOESM8_ESM.docx]

**S4 Table. Epistatic pairs**

Considering interaction terms between candidate SNPs (GWAS hits or eQTLs) for all combinations of genes with expression patterns associated either individually or in combination with MI in the Framingham Heart Study, we identify 5 instances of epistasis. Bootstrapped conditional regression was performed considering the main effect of each SNP and an interaction term. Reported is the percent of significant (p < 0.05) permutations out of 5000 for each term.

| #pair 1: VAMP8\|PHACTR1 | | | |
| --- | --- | --- | --- |
| GWAS Hits | rs1010 | rs9369640 | rs1010*rs9369640 |
|  | 6% | 1% | 7% |
| eQTLs | rs1009 | rs7774863 | rs1009*rs774863 |
|  | 20% | 9% | 47% |
| eQTLs | rs1009 | rs381134 | rs1009*rs381134 |
|  | 19% | 8% | 47% |
| eQTLs | rs1009 | rs2439538 | rs1009*rs2439538 |
|  | 14% | 8% | 39% |
| eQTLs | rs1010 | rs7774863 | rs1010*rs774863 |
|  | 21% | 9% | 48% |
| eQTLs | rs1010 | rs381134 | rs1010*rs381134 |
|  | 20% | 8% | 47% |
| eQTLs | rs1010 | rs2439538 | rs1010*rs2439538 |
|  | 15% | 9% | 43% |
| eQTLs | rs6757263 | rs7774863 | rs6757263*rs774863 |
|  | 21% | 10% | 55% |
| eQTLs | rs6757263 | rs381134 | rs6757263*rs381134 |
|  | 20% | 11% | 53% |
| eQTLs | rs6757263 | rs2439538 | rs6757263*rs2439538 |
|  | 15% | 11% | 45% |
| #pair 2: VAMP8\|ZEB2 | | | |
| GWAS Hits | rs1010 | rs17514846 | rs1010*rs17514846 |
|  | 20% | 29% | 25% |
| eQTLs | rs1009 | rs2677737 | rs1009*rs2677737 |
|  | 1% | 18% | 1% |
| eQTLs | rs1010 | rs2677737 | rs1010*rs2677737 |
|  | 0% | 17% | 1% |
| eQTLs | rs6757263 | rs2677737 | rs6757263*rs2677737 |
|  | 0% | 21% | 1% |
| #pair 3: PHACTR1\|FES | | | |
| GWAS Hits | rs9369640 | rs17514846 | rs9369640*rs17514846 |
|  | 20% | 29% | 25% |
| eQTLs | rs7774863 | rs2677737 | rs7774863*rs2677737 |
|  | 0% | 0% | 0% |
| eQTLs | rs381134 | rs2677737 | rs381134*rs2677737 |
|  | 1% | 20% | 1% |
| eQTLs | rs2439538 | rs2677737 | rs2439538*rs2677737 |
|  | 2% | 7% | 1% |
| #pair 4: VAMP8\|FES | | | |
| GWAS Hits | rs1010 | rs17514846 | rs1010*rs17514846 |
|  | 20% | 29% | 25% |
| eQTLs | rs1009 | rs2677737 | rs1009*rs2677737 |
|  | 1% | 18% | 1% |
| eQTLs | rs1010 | rs2677737 | rs1010*rs2677737 |
|  | 0% | 17% | 1% |
| eQTLs | rs6757263 | rs2677737 | rs6757263*rs2677737 |
|  | 0% | 21% | 1% |
| #pair 5: MIA3\|GUCY1A3 | | | |
| GWAS Hits | rs17465637 | rs7692387 | rs17465637*rs7692387 |
|  | 48% | 8% | 32% |
