# Supplementary material for: Non-linear interactions between candidate genes of myocardial infarction revealed in mRNA expression profiles
Source: BMC Genomics. 2016 Sep 17;17:738. doi: 10.1186/s12864-016-3075-6 (PMC5027110; doi:10.1186/s12864-016-3075-6)
Supplement: Additional file 10: — Differentially expressed candidate gene pairs in hypercholesterolemia. Connected graph formed by pairs of genes with a significant interaction term in CATHGEN using a logistic model accounting for MI (A) or hypercholesterolemia (B) with expression of gene 1, expression of gene 2, expression of gene 1 * expression of gene 2, and additional covariates (age, race, gender). C. Intersection of graphs for MI and hypercholesterolemia. Pink nodes indicate genes that are individually significant in MI (see Additional file 9) and orange nodes indicate genes that are individually significant in hypercholesterolemia (see Additional file 9). (PDF 1681 kb) [file 12864_2016_3075_MOESM10_ESM.pdf]

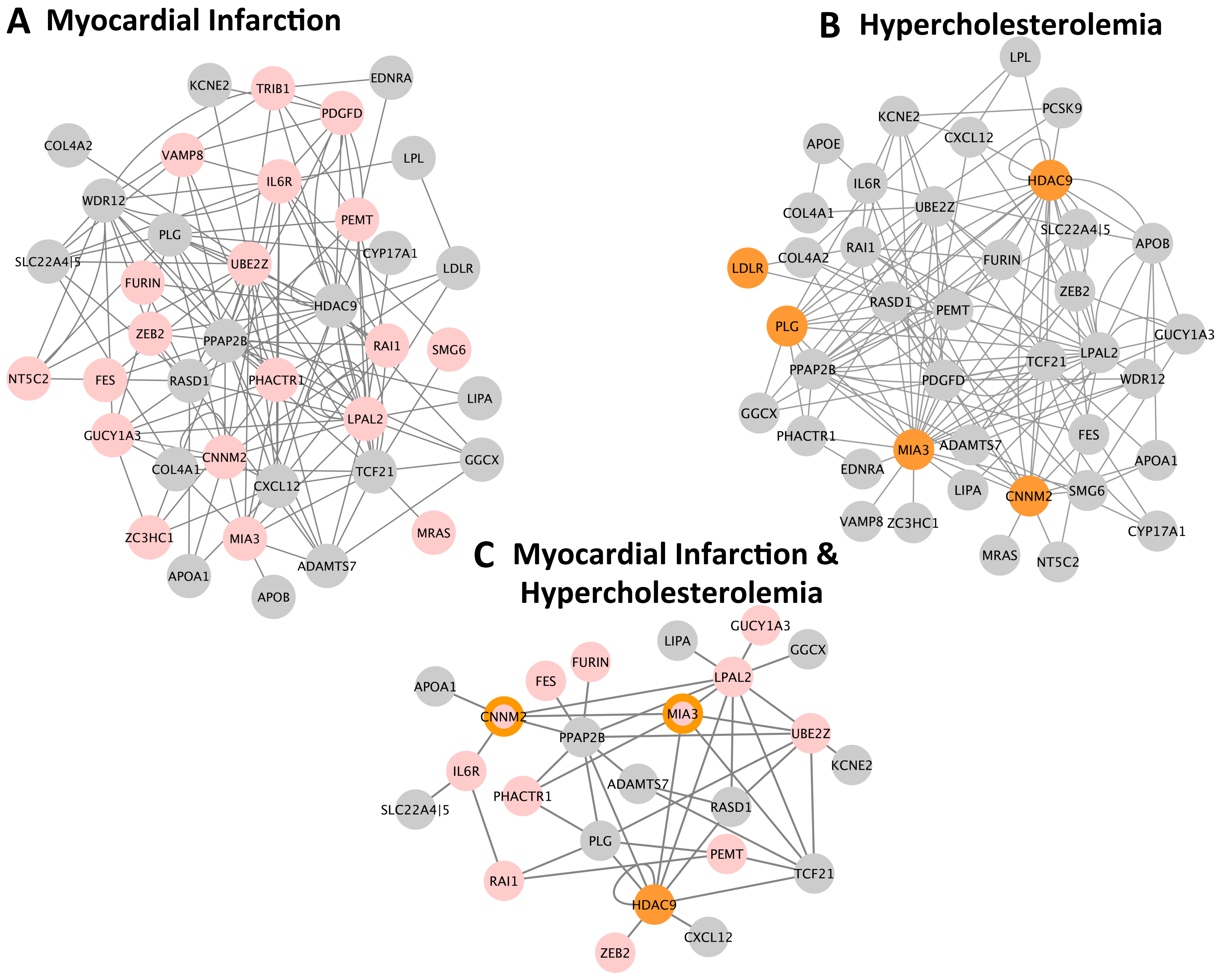
A Myocardial Infarction
KCNE2
TRIB1
EDNRA
PDGFD
COL4A2
VAMP8
IL6R
LPL
WDR12
PEMT
PLG
CYP17A1
SLC22A4|5
UBE2Z
LDLR
FURIN
HDAC9
ZEB2
PPAP2B
RAI1
SMG6
NT5C2
FES
RASD1
PHACTR1
LIPA
LPAL2
GUCY1A3
CNNM2
COL4A1
TCF21
GGCX
CXCL12
ZC3HC1
MIA3
MRAS
ADAMTS7
APOA1
APOB
B Hypercholesterolemia
LPL
PCSK9
KCNE2
CXCL12
APOE
HDAC9
IL6R
UBE2Z
COL4A1
SLC22A4|5
APOB
RAI1
COL4A2
FURIN
LDLR
ZEB2
RASD1
PEMT
PLG
GUCY1A3
LPAL2
TCF21
PPAP2B
PDGFD
WDR12
GGCX
PHACTR1
FES
MIA3
ADAMTS7
APOA1
EDNRA
LIPA
CNNM2
SMG6
VAMP8
ZC3HC1
CYP17A1
MRAS
NT5C2
C Myocardial Infarction &
Hypercholesterolemia
GUCY1A3
LIPA
GGCX
FURIN
FES
LPAL2
APOA1
CNNM2
MIA3
PPAP2B
UBE2Z
KCNE2
IL6R
ADAMTS7
PHACTR1
RASD1
SLC22A4|5
PLG
PEMT
TCF21
RAI1
HDAC9
CXCL12
ZEB2
