# Supplementary material for: Non-linear interactions between candidate genes of myocardial infarction revealed in mRNA expression profiles
Source: BMC Genomics. 2016 Sep 17;17:738. doi: 10.1186/s12864-016-3075-6 (PMC5027110; doi:10.1186/s12864-016-3075-6)
Supplement: Additional file 12: — Timing of MI relative to measure of gene expression in FHS. (DOCX 49 kb) [file 12864_2016_3075_MOESM12_ESM.docx]

| Number of events | Generation | Exam 1 | Average time of event (yrs since exam 1)* | Average time of event (estimated date) | Time of expression measure |
| --- | --- | --- | --- | --- | --- |
| 874 | 0: original cohort | 1948-1953 | 25 | 1973-1978 | 2005-2008 |
| 1281 | 1: offspring | 1971-1975 | 22 | 1993-1997 | 2005-2008 |
| 7 | 2: new offspring spouse | 2003-2005 | -7 | 1996-1998 | 2005-2008 |
| 146 | 3: generation 3 | 2002-2005 | -1 | 2001-2004 | 2005-2008 |
